# Serum Creatinine Clearance and Cognitive Function in Older Adults: Mediating Effects of Systemic Inflammatory Response Index

**DOI:** 10.1002/brb3.71413

**Published:** 2026-04-22

**Authors:** Yuyao He, Wenyue Hu, Cheu Hiutung, Zhenyun Han

**Affiliations:** ^1^ Shenzhen Hospital Beijing University of Chinese Medicine Shenzhen China; ^2^ Beijing University of Chinese Medicine Beijing China; ^3^ Dongfang Hospital Beijing University of Chinese Medicine Beijing China

**Keywords:** causal mediation analysis, cognitive function, geriatrics, NHANES, serum creatinine clearance, systemic Inflammatory Response Index (SIRI)

## Abstract

**Background:**

The association between renal dysfunction and cognitive decline in older adults has been increasingly recognized, although the underlying mechanisms remain poorly understood. This study explored the association between serum creatinine clearance (Ccr) and cognitive function, and evaluated whether the Systemic Inflammatory Response Index (SIRI) mediates this relationship.

**Methods:**

We analyzed 2482 adults aged ≥ 60 years from the 2011–2014 National Health and Nutrition Examination Survey (NHANES) using survey‐weighted regression models. Cognitive function was assessed using the Consortium to Establish a Registry for Alzheimer's Disease (CERAD) Word List Memory Test, Animal Fluency Test (AFT), Digit Symbol Substitution Test (DSST), and global cognitive performance (Z‐GCP). Associations among Ccr, SIRI, and cognitive outcomes were evaluated using regression models, subgroup and interaction analyses, restricted cubic splines, and mediation analysis with 10,000 bootstrap samples.

**Results:**

Higher Ccr was independently associated with better cognitive performance across all domains. In fully adjusted models, each unit increase in Ccr was significantly associated with higher cognitive scores (CERAD: *β* = 0.021, 95% CI: 0.010–0.032; AFT: *β* = 0.014, 95% CI: 0.003–0.025; DSST: *β* = 0.082, 95% CI: 0.029–0.134; Z‐GCP: *β* = 0.003, 95% CI: 0.002–0.005; all *p* < 0.05). Participants in the highest Ccr tertile had better cognitive performance and lower odds of impairment, especially in CERAD, DSST, and Z‐GCP (*p*‐trend < 0.05). Receiver operating characteristic analysis showed moderate discriminative ability of Ccr for cognitive impairment (AUCs: 0.736–0.843), and restricted cubic spline analyses revealed significant nonlinear associations for CERAD and DSST domains (*p* for nonlinearity < 0.05). Subgroup analyses indicated effect modification by age, sex, diabetes, hypertension, and stroke (*p*‐interaction < 0.05). Mediation analysis showed that SIRI partially mediated the association between Ccr and cognitive outcomes, accounting for 3%–6.6% of the mediated proportions in CERAD, DSST, and Z‐GCP domains (all *p* < 0.05).

**Conclusion:**

Ccr is independently associated with cognitive performance in older adults, particularly in the CERAD, DSST, and Z‐GCP domains. Systemic inflammation, as indicated by the SIRI, partially mediates these associations.

## Graphical abstract

Higher serum creatinine clearance (Ccr) is independently associated with cognitive performance across CERAD, DSST, and Z‐GCP domains in older adults, a relationship partially mediated by the Systemic Inflammatory Response Index (SIRI). This highlights a critical “kidney‐inflammation‐brain” axis, suggesting that preserving renal function and controlling inflammation are key strategies for cognitive health.

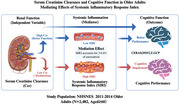

## 1 Introduction

Cognitive impairment and chronic kidney disease (CKD) are prevalent conditions that often co‐occur in older adults, each significantly contributing to increased morbidity, mortality, and a diminished quality of life (Jha et al. 2013; Pei et al. 2025; J. Zhang et al. 2024). Epidemiological studies have consistently demonstrated a strong association between impaired renal function and an elevated risk of cognitive decline, dementia, and related neuropsychiatric disorders (Davey et al. 2013; Kelly et al. 2024; Chi et al. 2023). Serum creatinine clearance (Ccr), a key marker of renal function, is widely used in clinical and epidemiological studies to assess kidney health, particularly in aging populations (Shahbaz et al. 2024; Drenth‐Van Maanen et al. 2013).

Unlike simple serum creatinine levels, Ccr integrates variables such as age, sex, and body weight, providing a more dynamic and accurate estimate of the kidney filtration capacity (Cockcroft and Gault 1976; Bjornsson 1979). A decline in Ccr signifies not only reduced fluid filtration but also the pathological retention of metabolic waste and uremic toxins, which disrupts systemic homeostasis and precipitates vascular endothelial dysfunction (Etgen et al. 2009; Arefin et al. 2025; Khatri et al. 2009). In the context of the “kidney–brain” axis, these systemic changes may compromise blood–brain barrier (BBB) integrity, potentially serving as a catalyst for neurodegeneration (Khatri et al. 2007). However, the precise pathophysiological mediators translating this renal decline into neural damage remain incompletely understood.

Recent mechanistic insights suggest that systemic inflammation serves as a critical bridge in this “kidney–brain” axis (Bhatraju et al. 2024; Kurella Tamura et al. 2017). As renal function declines, the accumulation of uremic toxins induces cytotoxic effects, triggering a persistent state of low‐grade inflammation (Brennan et al. 2021; Fu et al. 2022). The Systemic Inflammatory Response Index (SIRI), a composite biomarker integrating neutrophil, monocyte, and lymphocyte counts, offers a comprehensive assessment of the host's immune‐inflammatory balance (Li et al. 2024; Chu et al. 2024). Elevated SIRI levels have been independently associated with both renal insufficiency and cognitive decline (Gu et al. 2024; X. Wang et al. 2025), with specific correlations to oxidative stress and neuroinflammation, which may contribute to BBB dysfunction and neuronal apoptosis (Valibeygi et al. 2025). This suggests that renal dysfunction, as reflected by Ccr, triggers an inflammatory cascade, captured by SIRI, which subsequently exacerbates cognitive decline.

Despite these mechanistic insights, population‐based evidence quantifying the mediating role of SIRI in the Ccr–cognition relationship is limited. Using nationally representative data from the National Health and Nutrition Examination Survey (NHANES), this study aims to explore the association between serum Ccr and cognitive function in older adults, specifically testing the hypothesis that SIRI mediates this relationship. The findings may inform early risk stratification and the development of targeted therapeutic strategies for cognitive preservation in individuals with impaired renal function.

## 2 Methods

### 2.1 Data Sources and Study Population

Data for this study were derived from the 2011–2014 NHANES. NHANES utilizes a complex, multistage, stratified probability sampling design to ensure that the study sample is representative of the noninstitutionalized civilian population of the United States (Johnson et al. 2014)^.^ All NHANES data and documentation are publicly available at https://wwwn.cdc.gov/nchs/nhanes/. The NHANES study protocol received approval from the Ethics Review Board of the National Center for Health Statistics (NCHS), and all participants provided written informed consent.

A total of 19,931 participants were initially enrolled in the survey. Based on predefined inclusion and exclusion criteria, 2482 older adults aged 60 years or older were selected as the final analytic cohort. The specific exclusion criteria were as follows: (1) aged below 60 years or missing data on cognitive function scores (*n* = 16,997); (2) missing data on Ccr (*n* = 194); (3) missing data on the SIRI (*n* = 9); and (4) missing information on key covariates (*n* = 249).

### 2.2 Ccr Calculation

Ccr was calculated using the Cockcroft–Gault equation based on NHANES laboratory data. For males, the formula was as follows:

$$\mathrm{Ccr}=[(140-\mathrm{age})\times \mathrm{weight}\left(\mathrm{kg}\right)]/[72\times \mathrm{serum}\mathrm{creatinine}\left(\mathrm{mg}/\mathrm{dL}\right)]$$

For female participants, the calculated value was multiplied by 0.85 to account for sex‐specific differences in muscle mass. Among the 2482 participants, Ccr values ranged from 0.42 to 17.42 mg/dL (37.13–1539.04 µmol/L), and body weights ranged from 32.3 to 187.7 kg. For subsequent analyses, participants were categorized into tertiles based on their Ccr values as follows: Tertile 1 (T1), Ccr < 65.52 mL/min; Tertile 2 (T2), 65.52 ≤ Ccr < 87.52 mL/min; and Tertile 3 (T3), Ccr ≥ 87.52 mL/min.

### 2.3 Cognitive Function Assessment

Cognitive function was assessed using three widely recognized neuropsychological tests: the Consortium to Establish a Registry for Alzheimer's Disease (CERAD) Word Learning subtest, the Animal Fluency Test (AFT), and the Digit Symbol Substitution Test (DSST) (Xu et al. 2024). These tests evaluate distinct cognitive domains, including verbal memory, semantic fluency, processing speed, and executive function.

The CERAD assessment consists of two subtests: the Immediate Recall Test (IRT) and the Delayed Recall Test (DRT), which assess immediate and delayed verbal memory, respectively (Morris et al. 1989). In the IRT, participants are presented with a list of 10 unrelated words, which they are asked to recall immediately over the course of three learning trials. For each trial, the words are presented in a different randomized order. The IRT score, which ranges from 0 to 30, is calculated as the total number of words recalled correctly across all three trials. In the DRT, after a short interval, participants are asked to recall the same list of words. The number of correct responses forms the DRT score, which ranges from 0 to 10. The overall CERAD score is obtained by summing the IRT and DRT scores, with a maximum possible score of 40 (X. Wang, Xiao, et al. 2022).

The AFT is designed to assess both semantic memory and verbal fluency (Song et al. 2023). During the assessment, participants are instructed to name as many different animals as possible within a 60‐s interval. The total number of unique, correct animal names generated constitutes the AFT score, which ranges from 0 to 50, with higher scores reflecting greater verbal fluency. This test is widely utilized in both clinical practice and large‐scale epidemiological studies investigating cognitive aging.

The DSST, a component of the Wechsler Adult Intelligence Scale, is employed to evaluate processing speed, attention, and executive function (Wu et al. 2023). Participants receive a key that pairs the digits 1 through 9 with corresponding symbols. They are instructed to match as many symbols to their corresponding digits as possible within a 120‐s interval. The total number of correct symbol‐digit pairings constitutes the DSST score, which ranges from 0 to 133.

To derive a composite measure of global cognitive performance, raw scores from the CERAD, AFT, and DSST were standardized to *Z*‐scores using the mean and standard deviation of the study population. These *Z*‐scores were subsequently averaged to generate a global cognitive performance (GCP) *Z*‐score, which reflects the overall cognitive function of each participant (Shi et al. 2023).

At present, no universally accepted diagnostic threshold or gold standard exists for defining poor cognitive function using these three neuropsychological assessments alone. Consistent with established epidemiological research and in light of the lack of universally recognized cutoff values for cognitive impairment, individuals with test scores in the lowest quartile were classified as cognitively impaired, in accordance with previous studies (S. P. Chen et al. 2017; X. Dong et al. 2020; Peeri et al. 2021). The first‐quartile (Q1) thresholds used in this analysis were 21 for the CERAD, 13 for the AFT, 34 for the DSST, and −0.54 for the composite GCP *Z*‐score.

### 2.4 Calculation of SIRI

All hematological parameters were measured at the NHANES Mobile Examination Center utilizing standardized laboratory protocols. Neutrophil, lymphocyte, and monocyte counts were determined with a Beckman Coulter HMX hematology analyzer and expressed in units of ×10^3^/µL. The SIRI was calculated according to the formula: SIRI = (monocyte count × neutrophil count)/lymphocyte count (Zheng et al. 2024). For analytical purposes, participants were divided into tertiles based on SIRI values: T1 (0.06 ≤ SIRI < 0.87), T2 (0.87 ≤ SIRI < 1.46), and T3 (1.46 ≤ SIRI ≤ 24.60).

### 2.5 Covariates

Consistent with prior literature, we adjusted for a comprehensive set of covariates associated with Ccr, cognitive outcomes, and SIRI to minimize potential confounding. Demographic variables included sex (male or female), age (continuous), and race/ethnicity (non‐Hispanic White, non‐Hispanic Black, Mexican American, or other) (Wu et al. 2023), as well as educational attainment, which was categorized as less than high school, high school or general educational development (GED), and college or above. Marital status was classified as either married or cohabiting, or unmarried/not partnered, with the latter group comprising individuals who were widowed, divorced, separated, or never married (Z. F. Wang et al. 2021). Socioeconomic status was assessed using the poverty income ratio (PIR), where values below 1.0 indicated household income beneath the federal poverty threshold, and values of 1.0 or greater represented income at or above this threshold (Wei et al. 2023).

Anthropometric measures encompassed body mass index (BMI), which was categorized as < 25, 25–29.9, and ≥ 30 kg/m^2^ (Casagrande et al. 2021). Medical comorbidities assessed in this study included diabetes mellitus, hypertension, cardiovascular disease (CVD), and stroke. Diabetes mellitus was defined as a self‐reported physician diagnosis, current use of insulin or oral hypoglycemic agents, or laboratory evidence, including fasting plasma glucose ≥ 7.0 mmol/L, 2‐h plasma glucose ≥ 11.1 mmol/L after an oral glucose tolerance test, random plasma glucose ≥ 11.1 mmol/L, or glycated hemoglobin (HbA1c) ≥ 6.5% (G. Dong et al. 2023). Hypertension was identified by self‐reported physician diagnosis, current use of antihypertensive medication, systolic blood pressure ≥ 140 mmHg, or diastolic blood pressure ≥ 90 mmHg (Yuan et al. 2024). CVD was defined as a self‐reported history of coronary heart disease, angina pectoris, myocardial infarction, or heart attack (Casagrande et al. 2021). Stroke status was determined according to self‐reported physician diagnosis.

### 2.6 Statistical Analyses

All analyses were performed using R software (version 4.4.2), with NHANES sampling weights applied to address the complex, multistage, stratified cluster sampling design. Continuous variables were expressed as weighted means with standard deviations, while categorical variables were summarized as weighted percentages. To evaluate group differences, the Kruskal–Wallis test was applied to continuous variables, whereas the chi‐square test was employed for categorical variables.

The associations between Ccr and cognitive performance were assessed using generalized linear models, with sequential covariate adjustment implemented as follows: Model 1 (unadjusted), Model 2 (adjusted for demographic factors), and Model 3 (further adjusted for covariates described in Section 2.5). Linear trend tests were conducted across tertiles of Ccr. To explore potential effect modification, subgroup analyses and multiplicative interaction terms were incorporated. The robustness of the findings was further evaluated using multivariate logistic regression to examine the association between Ccr and the risk of cognitive impairment. Receiver operating characteristic (ROC) curves and the corresponding area under the curve (AUC) values were generated to assess model discrimination. Restricted cubic spline (RCS) regression analyses were conducted to examine potential nonlinear associations.

Causal mediation analysis was conducted using the “mediation” package in R, employing a nonparametric bootstrap approach with 10,000 quasi‐Bayesian Monte Carlo simulations to estimate the mediating effect of the SIRI on the association between Ccr and cognitive outcomes. The mediation effect was quantified as the average causal mediation effect (ACME), average direct effect (ADE), total effect, and proportion mediated, with adjustment for the same covariates described above. Specifically, three effect pathways were considered: (1) the direct effect of Ccr on cognitive outcomes (Ccr → cognition); (2) the indirect (mediated) effect of Ccr on cognition through SIRI (Ccr → SIRI → cognition); (3) the total effect, which represents the combined impact of both the direct and indirect pathways. A two‐sided *p*‐value < 0.05 was considered statistically significant.

## 3 Results

### 3.1 Association Between SIRI and Cognitive Function

A total of 2482 adults aged 60 years and older were included in the analysis (Table 1). Stratified analyses by tertiles of Ccr revealed that participants in the lowest Ccr group were older, more likely to be female, widowed, divorced, separated, or never married, and exhibited a lower BMI. Additionally, these individuals had a higher prevalence of hypertension, CVD, and stroke (all *p* < 0.05). Notably, this study population exhibited a high educational attainment, with approximately 63% having a college education or higher, substantially exceeding the national averages for US adults born between the 1930s and 1950s.

**TABLE 1:** Baseline characteristics.

Characteristic	*N*	Overall	T1	T2	T3	*p*‐value
Age (years)	2482					< 0.001
60–70		1469 (62%)	254 (30%)	536 (66%)	679 (83%)	
70–80		1013 (38%)	573 (70%)	291 (34%)	149 (17%)	
Sex	2482					< 0.001
Male		1212 (46%)	361 (36%)	416 (48%)	435 (53%)	
Female		1270 (54%)	466 (64%)	411 (52%)	393 (47%)	
Race/ethnicity, *n* (%)	2482					0.015
Mexican American		213 (3.2%)	42 (2.3%)	63 (2.8%)	108 (4.3%)	
Non‐Hispanic White		1241 (81%)	461 (80%)	395 (80%)	385 (82%)	
Non‐Hispanic Black		562 (7.7%)	183 (8.9%)	181 (7.6%)	198 (7.0%)	
Other		466 (8.1%)	141 (8.6%)	188 (9.5%)	137 (6.4%)	
Educational level, *n* (%)	2482					0.059
Less than high school		261 (5.3%)	93 (7.0%)	91 (5.3%)	77 (3.9%)	
High school or GED		921 (32%)	319 (35%)	308 (31%)	294 (30%)	
College or above		1300 (63%)	415 (58%)	428 (64%)	457 (66%)	
Marital status, *n* (%)	2482					0.001
Married or living with partner		1441 (65%)	425 (57%)	497 (69%)	519 (68%)	
Unmarried/not partnered		1041 (35%)	402 (43%)	330 (31%)	309 (32%)	
PIR	2482					0.3
< 1		408 (8.8%)	137 (10%)	130 (7.8%)	141 (8.7%)	
≥ 1		2074 (91%)	690 (90%)	697 (92%)	687 (91%)	
BMI (kg/m^2^)	2482					< 0.001
< 25		658 (26%)	380 (49%)	220 (28%)	58 (5.3%)	
25–30		879 (37%)	292 (34%)	351 (45%)	236 (32%)	
≥ 30		945 (38%)	155 (18%)	256 (27%)	534 (63%)	
Diabetes	2482					0.004
Yes		573 (19%)	198 (19%)	164 (15%)	211 (23%)	
No		1909 (81%)	629 (81%)	663 (85%)	617 (77%)	
Hypertension	2482					0.003
Yes		1551 (59%)	559 (64%)	474 (52%)	518 (60%)	
No		931 (41%)	268 (36%)	353 (48%)	310 (40%)	
CVD	2482					0.02
Yes		431 (18%)	198 (22%)	124 (17%)	109 (15%)	
No		2051 (82%)	629 (78%)	703 (83%)	719 (85%)	
Stroke	2482					0.009
Yes		169 (6.2%)	81 (9.1%)	57 (6.3%)	31 (3.8%)	
No		2313 (94%)	746 (91%)	770 (94%)	797 (96%)	

*Note*: Continuous variables are expressed as mean ± standard deviation (SD), and *p*‐values were calculated using the Kruskal–Wallis *H* test. Categorical variables are expressed as survey‐weighted percentages, with *p*‐values calculated using the chi‐square test. Ccr tertiles were defined as follows: Tertile 1 (T1), Ccr < 65.52 mL/min; Tertile 2 (T2), 65.52 ≤ Ccr < 87.52 mL/min; and Tertile 3 (T3), Ccr ≥ 87.52 mL/min.

Abbreviations: BMI, body mass index; CVD, cardiovascular disease; GED, general educational development; PIR, poverty income ratio.

### 3.2 Multivariable Linear Regression Analysis of Ccr and Cognitive Function

Multivariable linear regression analyses demonstrated that higher Ccr was independently associated with better cognitive performance across all domains (CERAD, AFT, DSST, Z‐GCP) in older adults (Table 2). In fully adjusted models, each unit increase in Ccr was significantly associated with higher scores for CERAD (*β* = 0.021, 95% CI: 0.010–0.032, *p* = 0.001), AFT (*β* = 0.014, 95% CI: 0.003–0.025, *p* = 0.017), DSST (*β* = 0.082, 95% CI: 0.029–0.134, *p* = 0.005), and Z‐GCP (*β* = 0.003, 95% CI: 0.002–0.005, *p* < 0.001). Participants in the highest Ccr tertile had significantly better cognitive scores than those in the lowest tertile, with significant linear trends across all domains (all *p*‐trend ≤ 0.05). These associations persisted after comprehensive adjustment for demographic, socioeconomic, and clinical covariates.

**TABLE 2:** Multivariable linear regression analysis of Ccr and cognitive function in older adults.

Outcomes	Ccr	Model 1 *β* (95% CI)	Model 2 *β* (95% CI)	Model 3 *β* (95% CI)
CERAD	Continuous	0.042 (0.030, 0.055)	0.018 (0.007, 0.028)	0.021 (0.010, 0.032)
	*p*‐value	< 0.001	0.002	0.001
	T1	Ref	Ref	Ref
	T2	2.332 (1.507, 3.157)	1.122 (0.243, 2.001)	1.162 (0.271, 2.053)
	T3	3.203 (2.310, 4.096)	1.438 (0.650, 2.226)	1.664 (0.779, 2.550)
	*p*‐trend	< 0.001	0.001	0.001
AFT	Continuous	0.036 (0.026, 0.047)	0.010 (0.001, 0.019)	0.014 (0.003, 0.025)
	*p*‐value	< 0.001	0.025	0.017
	T1	Ref	Ref	Ref
	T2	1.779 (1.064, 2.493)	0.579 (−0.225, 1.383)	0.641 (−0.205, 1.486)
	T3	2.929 (2.274, 3.584)	1.139 (0.487, 1.790)	1.466 (0.573, 2.360)
	*p*‐trend	< 0.001	0.001	0.003
DSST	Continuous	0.146 (0.104, 0.188)	0.067 (0.026, 0.107)	0.082 (0.029, 0.134)
	*p*‐value	< 0.001	0.002	0.005
	T1	Ref	Ref	Ref
	T2	6.749 (4.766, 8.732)	2.739 (0.819, 4.659)	2.454 (0.623, 4.284)
	T3	10.194 (7.522, 12.866)	4.360 (2.081, 6.639)	4.716 (1.794, 7.637)
	*p*‐trend	< 0.001	0.001	0.004
Z‐GCP	Continuous	0.007 (0.005, 0.008)	0.003 (0.002, 0.004)	0.003 (0.002, 0.005)
	*p*‐value	< 0.001	< 0.001	< 0.001
	T1	Ref	Ref	Ref
	T2	0.346 (0.251, 0.441)	0.147 (0.041, 0.252)	0.147 (0.046, 0.247)
	T3	0.510 (0.400, 0.620)	0.218 (0.124, 0.312)	0.250 (0.139, 0.361)
	*p*‐trend	< 0.001	< 0.001	< 0.001

*Note*: T1: Ccr < 65.52 mL/min, T2: 65.52 ≤ Ccr < 87.52 mL/min, and T3: Ccr ≥ 87.52 mL/min. Model 1: Unadjusted analyses. Model 2: Adjusted for sex, age, race/ethnicity, educational level, marital status, and poverty income ratio. Model 3: Adjusted for all covariates in Model 2, with additional adjustment for BMI, diabetes, hypertension, cardiovascular disease, and stroke.

Abbreviations: AFT, Animal Fluency Test; Ccr, creatinine clearance; CERAD, Consortium to Establish a Registry for Alzheimer's Disease; CI, confidence interval; DSST, Digit Symbol Substitution Test; GCP, global cognitive performance.

### 3.3 Multivariable Logistic Regression and Predictive Performance

Multivariable logistic regression analyses (Table 3) indicated that higher Ccr was independently associated with reduced odds of cognitive impairment in both CERAD (OR = 0.989, 95% CI: 0.982–0.996, *p* = 0.006) and Z‐GCP (OR = 0.984, 95% CI: 0.977–0.991, *p* < 0.001), after adjustment for potential confounders. In tertile analyses, participants in the highest Ccr group exhibited significantly lower odds of cognitive impairment in CERAD, DSST, and Z‐GCP compared to those in the lowest group, with significant trends observed across tertiles (all *p*‐trend < 0.05). No significant association was observed for AFT (*p* = 0.275, *p*‐trend = 0.251).

**TABLE 3:** Multivariable logistic regression analysis between Ccr and cognitive function.

Outcomes	Ccr	Model 1 OR (95% CI)	Model 2 OR (95% CI)	Model 3 OR (95% CI)
CERAD	Continuous	0.981 (0.975, 0.986)	0.988 (0.982, 0.994)	0.989 (0.982, 0.996)
	*p*‐value	< 0.001	< 0.001	0.006
	T1	Ref	Ref	Ref
	T2	0.444 (0.319, 0.616)	0.576 (0.395, 0.839)	0.610 (0.404, 0.921)
	T3	0.303 (0.201, 0.455)	0.466 (0.301, 0.721)	0.513 (0.300, 0.876)
	*p*‐trend	< 0.001	0.001	0.015
AFT	Continuous	0.985 (0.979, 0.990)	0.995 (0.989, 1.001)	0.996 (0.988, 1.004)
	*p*‐value	< 0.001	0.118	0.275
	T1	Ref	Ref	Ref
	T2	0.495 (0.356, 0.688)	0.718 (0.477, 1.081)	0.751 (0.482, 1.169)
	T3	0.359 (0.257, 0.500)	0.671 (0.419, 1.073)	0.693 (0.348, 1.379)
	*p*‐trend	< 0.001	0.084	0.251
DSST	Continuous	0.978 (0.970, 0.985)	0.986 (0.974, 0.999)	0.985 (0.970, 1.001)
	*p*‐value	< 0.001	0.037	0.066
	T1	Ref	Ref	Ref
	T2	0.426 (0.314, 0.578)	0.567 (0.375, 0.857)	0.591 (0.373, 0.937)
	T3	0.270 (0.201, 0.363)	0.451 (0.265, 0.767)	0.426 (0.211, 0.861)
	*p*‐trend	< 0.001	0.005	0.018
Z‐GCP	Continuous	0.975 (0.970, 0.981)	0.985 (0.978, 0.991)	0.984 (0.977, 0.991)
	*p*‐value	< 0.001	< 0.001	< 0.001
	T1	Ref	Ref	Ref
	T2	0.443 (0.313, 0.628)	0.622 (0.410, 0.944)	0.644 (0.423, 0.981)
	T3	0.245 (0.169, 0.355)	0.430 (0.287, 0.642)	0.437 (0.261, 0.730)
	*p*‐trend	< 0.001	< 0.001	0.003

*Note*: T1: Ccr < 65.52 mL/min, T2: 65.52 ≤ Ccr < 87.52 mL/min, and T3: Ccr ≥ 87.52 mL/min. Model 1: Unadjusted analyses. Model 2: Adjusted for sex, age, race/ethnicity, educational level, marital status, and poverty income ratio. Model 3: Adjusted for all covariates in Model 2, with additional adjustment for BMI, diabetes, hypertension, cardiovascular disease, and stroke.

Abbreviations: AFT, Animal Fluency Test; Ccr, creatinine clearance; CERAD, Consortium to Establish a Registry for Alzheimer's Disease; CI: confidence Interval; DSST, Digit Symbol Substitution Test; GCP, global cognitive performance; OR, odds ratios.

**FIGURE 1:**
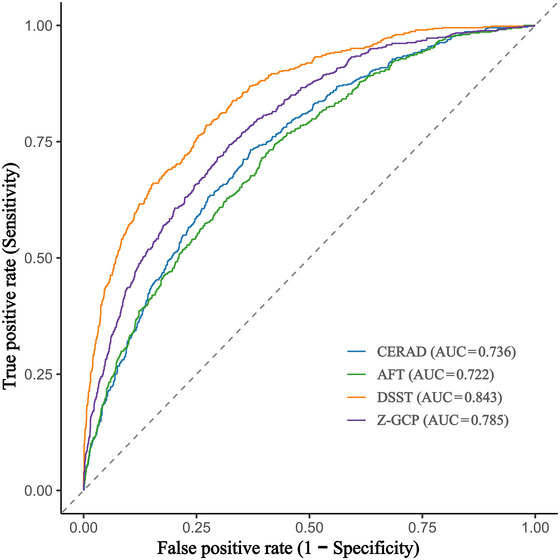
ROC curves of Ccr for predicting cognitive impairment across different cognitive domains. All analyses were adjusted for sex, age, race/ethnicity, educational level, marital status, poverty income ratio, BMI, diabetes, hypertension, cardiovascular disease, and stroke. AFT, Animal Fluency Test; AUC, area under the curve; CERAD, Consortium to Establish a Registry for Alzheimer's Disease; DSST, Digit Symbol Substitution Test; GCP, global cognitive performance.

**FIGURE 2:**
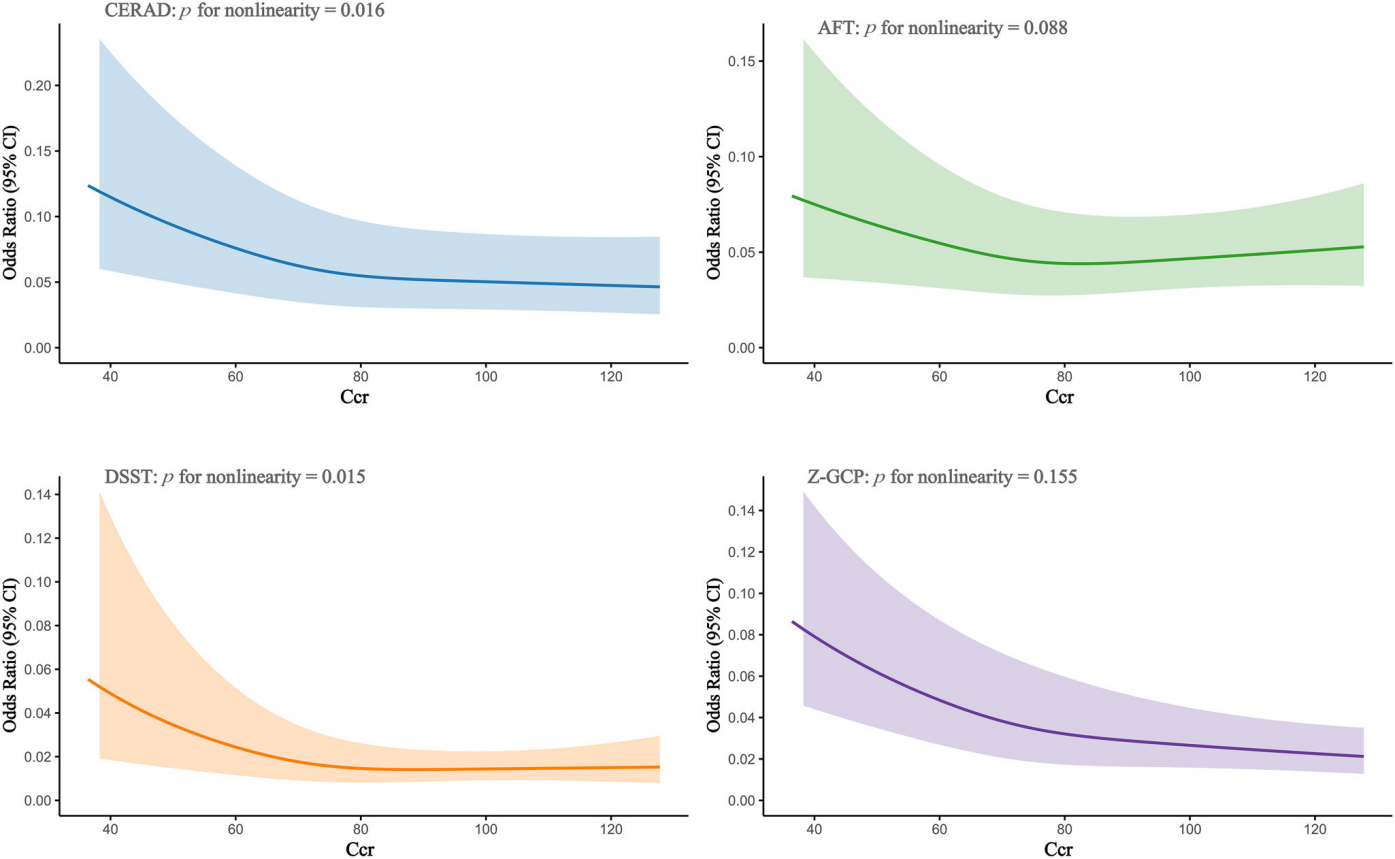
RCS analysis of the nonlinear associations between Ccr and cognitive impairment. All analyses were adjusted for sex, age, race/ethnicity, educational level, marital status, poverty income ratio, BMI, diabetes, hypertension, cardiovascular disease, and stroke.

ROC analysis demonstrated that Ccr provided moderate discrimination for cognitive impairment, with AUCs of 0.736 for CERAD, 0.722 for AFT, 0.843 for DSST, and 0.785 for Z‐GCP (all *p* < 0.001; Figure 1). Model calibration was acceptable for most cognitive domains, except for DSST. The optimal cutoff values derived from the Youden index resulted in sensitivities and specificities ranging from 0.576 to 0.784 (Table 4). Nonlinear associations between Ccr and cognitive function were further explored using RCS analysis (Figure 2). Significant nonlinear relationships were identified for the CERAD (*p* for nonlinearity = 0.016) and DSST (*p* for nonlinearity = 0.015) domains, with the lowest risk of cognitive impairment observed at Ccr values of 127.8 mL/min for CERAD and 88.3 mL/min for DSST, respectively. No significant nonlinear associations were observed for AFT (*p* for nonlinearity = 0.088) and Z‐GCP (*p* for nonlinearity = 0.155).

**TABLE 4:** Summary of ROC, model calibration, and optimal cutoff metrics for Ccr in predicting cognitive function.

Outcomes	ROC			Hosmer–Lemeshow			Youden		
	AUC	95% CI	*p*‐value	*χ* ^2^	df	*p*‐value	Cutoff	Specificity (%)	Sensitivity (%)
CERAD	0.736	(0.714, 0.758)	< 0.001	10.392	8.000	0.239	0.213	0.631	0.732
AFT	0.722	(0.698, 0.745)	< 0.001	13.720	8.000	0.089	0.170	0.576	0.745
DSST	0.843	(0.825, 0.860)	< 0.001	21.012	8.000	0.007	0.197	0.730	0.784
Z‐GCP	0.785	(0.765, 0.805)	< 0.001	13.049	8.000	0.110	0.210	0.678	0.744

*Note*: All analyses were adjusted for sex, age, race/ethnicity, educational level, marital status, poverty income ratio, BMI, diabetes, hypertension, cardiovascular disease, and stroke.

Abbreviations: AUC, area under the curve; CI, confidence Interval; ROC, receiver operating characteristic.

### 3.4 Subgroup Heterogeneity Analysis

**FIGURE 3:**
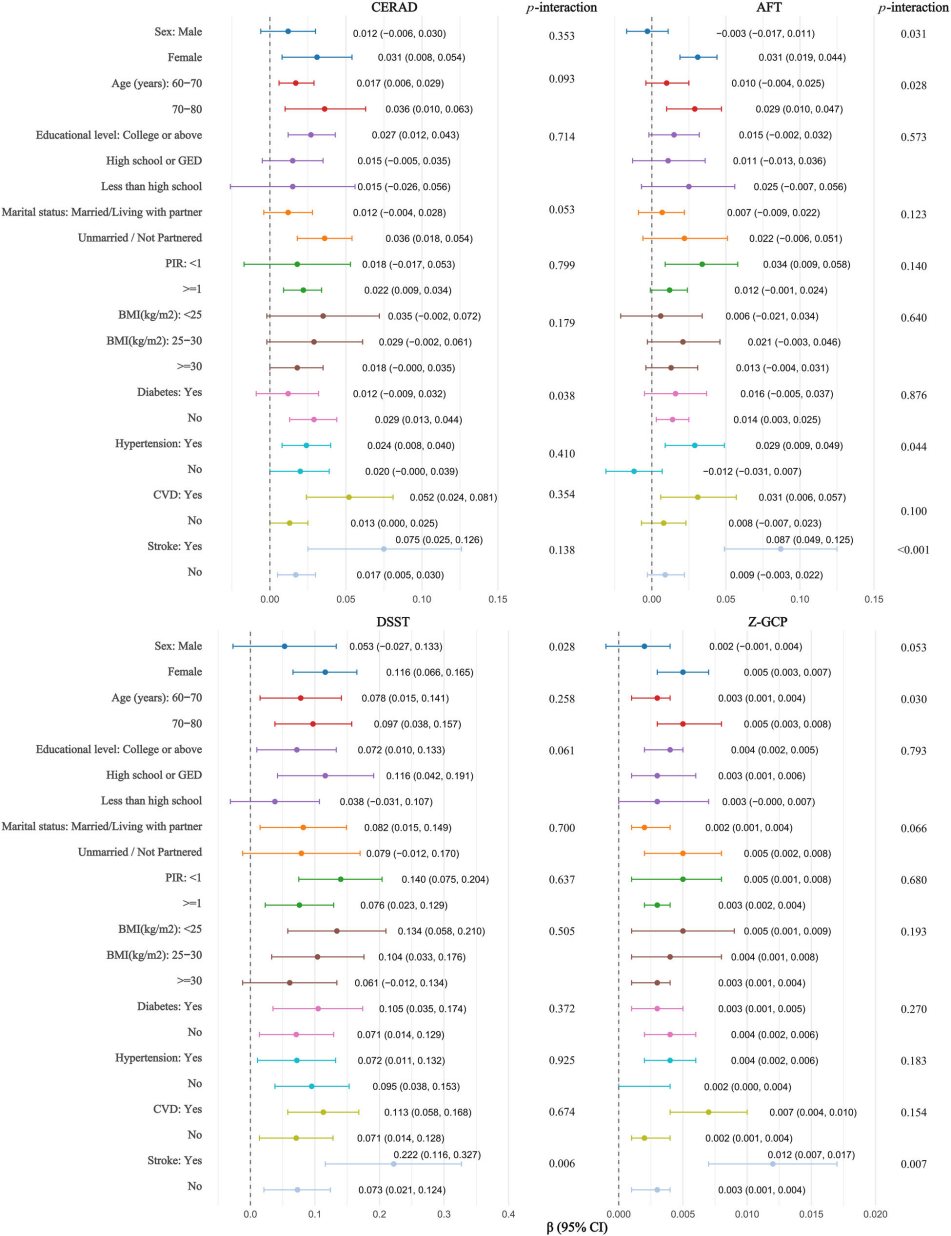
Subgroup analyses of Ccr and cognitive function. All analyses were adjusted for sex, age, race/ethnicity, educational level, marital status, poverty income ratio, BMI, diabetes, hypertension, cardiovascular disease, and stroke. AFT, Animal Fluency Test; BMI, body mass index; CERAD, Consortium to Establish a Registry for Alzheimer's Disease; CI, confidence interval; DSST, Digit Symbol Substitution Test; GCP, global cognitive performance; GED, general educational development; PIR, poverty income ratio.

Subgroup analyses (Figure 3) demonstrated that the positive association between Ccr and cognitive function persisted across most clinical and demographic strata, and several significant interactions were observed. The association was notably stronger and statistically significant in females, with sex specific effects evident for both AFT (*p*‐interaction = 0.031) and DSST (*p*‐interaction = 0.028). Participants aged 70–80 years exhibited more pronounced associations than those aged 60–70 years, particularly for AFT (*p*‐interaction = 0.028) and Z‐GCP (*p*‐interaction = 0.030). Diabetes status significantly modified the relationship with CERAD (*p*‐interaction = 0.038), with associations more apparent in nondiabetic individuals. Among hypertensive participants, the positive association with cognitive outcomes remained significant, and hypertension amplified the association with AFT (*p*‐interaction = 0.044). Individuals with a history of stroke demonstrated the strongest associations, with significant interactions for AFT (*p*‐interaction < 0.001), DSST (*p*‐interaction = 0.006), and Z‐GCP (*p*‐interaction = 0.007).

### 3.5 Ternary Relationship Between Ccr, SIRI, and Cognitive Function

#### 3.5.1 Association Between Ccr and SIRI

As shown in Table 5, a higher Ccr was significantly associated with lower SIRI values. In Model 3, each unit increase in Ccr was associated with a reduction in SIRI (*β* = −0.003, 95% CI: −0.005 to −0.002, *p* = 0.001). Compared to T1, T3 was associated with a significantly lower SIRI level (*β* = −0.252, 95% CI: −0.458 to −0.045), and a significant linear trend was observed across tertiles (*p*‐trend = 0.018). These associations remained robust after adjustment for all relevant covariates.

**TABLE 5:** Association between Ccr and SIRI.

Outcomes	Ccr		Model 1 *β* (95% CI)	Model 2 *β* (95% CI)	Model 3 *β* (95% CI)
SIRI	Continuous		−0.003 (−0.005, −0.001)	−0.002 (−0.003, −0.000)	−0.003 (−0.005, −0.002)
	*p*‐value		< 0.001	0.025	0.001
	T1		Ref	Ref	Ref
	T2		−0.227 (−0.393, −0.060)	−0.167 (−0.362, 0.027)	−0.157 (−0.355, 0.041)
	T3		−0.260 (−0.414, −0.107)	−0.180 (−0.377, 0.016)	−0.252 (−0.458, −0.045)
	*p*‐trend		0.002	0.074	0.018

*Note*: T1: Ccr < 65.52 mL/min, T2: 65.52 ≤ Ccr < 87.52 mL/min, and T3: Ccr ≥ 87.52 mL/min. Model 1: Unadjusted analyses. Model 2: Adjusted for sex, age, race/ethnicity, educational level, marital status, and poverty income ratio. Model 3: Adjusted for all covariates in Model 2, with additional adjustment for BMI, diabetes, hypertension, cardiovascular disease, and stroke.

Abbreviations: Ccr, creatinine clearance; CI, confidence interval; SIRI, Systemic Inflammatory Response Index.

#### 3.5.2 Association Between SIRI and Cognitive Function

In Model 3, higher SIRI was significantly associated with lower cognitive scores across multiple domains (Table 6). SIRI demonstrated significant inverse associations with CERAD (*β* = −0.352, 95% CI: −0.638 to −0.067, *p* = 0.019), AFT (*β* = −0.248, 95% CI: −0.486 to −0.011, *p* = 0.042), DSST (*β* = −0.780, 95% CI: −1.370 to −0.190, *p* = 0.013), and Z‐GCP (*β* = −0.051, 95% CI: −0.083 to −0.019, *p* = 0.004) when analyzed as continuous variables. Similar patterns emerged in tertile analyses, where participants in the highest SIRI tertile generally exhibited lower cognitive scores than those in the lowest tertile, with a significant linear trend for Z‐GCP (*p*‐trend = 0.048). Although some associations were attenuated after full adjustment, the overall findings indicate that higher SIRI is independently associated with poorer cognitive function.

**TABLE 6:** Association between SIRI and cognitive function.

Outcomes	SIRI	Model 1 *β* (95% CI)	Model 2 *β* (95% CI)	Model 3 *β* (95% CI)
CERAD	Continuous	−0.812 (−1.133, −0.490)	−0.408 (−0.676, −0.141)	−0.352 (−0.638, −0.067)
	*p*‐value	< 0.001	0.005	0.019
	T1	Ref	Ref	Ref
	T2	−0.751 (−1.801, 0.299)	0.030 (−0.889, 0.948)	0.106 (−0.868, 1.079)
	T3	−2.143 (−3.054, −1.232)	−0.863 (−1.736, 0.010)	−0.720 (−1.680, 0.240)
	*p*‐trend	< 0.001	0.041	0.107
AFT	Continuous	−0.385 (−0.684, −0.086)	−0.310 (−0.557, −0.064)	−0.248 (−0.486, −0.011)
	*p*‐value	0.013	0.016	0.042
	T1	Ref	Ref	Ref
	T2	0.378 (−0.416, 1.172)	0.498 (−0.126, 1.121)	0.586 (−0.090, 1.262)
	T3	−0.632 (−1.370, 0.106)	−0.424 (−1.007, 0.158)	−0.258 (−0.879, 0.363)
	*p*‐trend	0.066	0.095	0.288
DSST	Continuous	−1.912 (−2.694, −1.129)	−1.120 (−1.681, −0.559)	−0.780 (−1.370, −0.190)
	*p*‐value	< 0.001	< 0.001	0.013
	T1	Ref	Ref	Ref
	T2	−0.327 (−2.326, 1.672)	1.068 (−0.425, 2.560)	1.330 (−0.305, 2.964)
	T3	−4.819 (−7.313, −2.325)	−2.510 (−4.494, −0.525)	−1.715 (−3.882, 0.453)
	*p*‐trend	< 0.001	0.010	0.081
Z‐GCP	Continuous	−0.107 (−0.148, −0.065)	−0.063 (−0.093, −0.033)	−0.051 (−0.083, −0.019)
	*p*‐value	< 0.001	< 0.001	0.004
	T1	Ref	Ref	Ref
	T2	−0.042 (−0.167, 0.084)	0.039 (−0.058, 0.136)	0.052 (−0.054, 0.158)
	T3	−0.258 (−0.369, −0.147)	−0.125 (−0.215, −0.035)	−0.096 (−0.200, 0.009)
	*p*‐trend	< 0.001	0.005	0.048

*Note*: T1: 0.06 ≤ SIRI < 0.87, T2: 0.87 ≤ SIRI < 1.46, and T3: 1.46 ≤ SIRI ≤ 24.60. Model 1: Unadjusted analyses. Model 2: Adjusted for sex, age, race/ethnicity, educational level, marital status, and poverty income ratio. Model 3: Adjusted for all covariates in Model 2, with additional adjustment for BMI, diabetes, hypertension, cardiovascular disease, and stroke.

Abbreviations: AFT, Animal Fluency Test; Ccr, creatinine clearance; CERAD, Consortium to Establish a Registry for Alzheimer's Disease; CI, confidence interval; DSST, Digit Symbol Substitution Test; GCP, global cognitive performance.

#### 3.5.3 Mediating Effects of SIRI

Mediation analysis revealed that SIRI significantly mediated the association between Ccr and multiple cognitive domains (Table 7). For CERAD, DSST, and Z‐GCP scores, the mediated proportions were 6.6% (95% CI: 0.017–0.186, *p* = 0.002), 3% (95% CI: 0.003–0.075, *p* = 0.027), and 4.6% (95% CI: 0.014–0.103, *p* = 0.002), respectively. The ACME for these domains was also statistically significant (*p* < 0.05). No significant mediation was observed for AFT (*p* = 0.121). Across all domains, direct effects remained robust (*p* < 0.05).

**TABLE 7:** Mediation analysis of Ccr and cognitive function by SIRI.

	ACME *β* (95% CI)	ADE *β* (95% CI)	Total effect *β* (95% CI)	Mediated proportion *β* (95% CI)
CERAD	0.001 (0.000, 0.003)	0.018 (0.007, 0.029)	0.019 (0.008, 0.030)	0.066 (0.017, 0.186)
*p*‐value	0.002	0.001	< 0.001	0.002
AFT	0.001 (0.000, 0.002)	0.021 (0.011, 0.030)	0.021 (0.012, 0.031)	0.028 (−0.007, 0.092)
*p*‐value	0.121	< 0.001	< 0.001	0.121
DSST	0.002 (0.000, 0.005)	0.072 (0.048, 0.098)	0.075 (0.050, 0.100)	0.030 (0.003, 0.075)
*p*‐value	0.027	< 0.001	< 0.001	0.027
Z‐GCP	0.001 (0.000, 0.001)	0.003 (0.002, 0.005)	0.003(0.002, 0.005)	0.046 (0.014, 0.103)
*p*‐value	0.002	< 0.001	< 0.001	0.002

*Note*: All analyses were adjusted for sex, age, race/ethnicity, educational level, marital status, poverty income ratio, BMI, diabetes, hypertension, cardiovascular disease, and stroke.

Abbreviations: ACME, average causal mediation effect; ADE, average direct effect; AFT, Animal Fluency Test; CERAD, Consortium to Establish a Registry for Alzheimer's Disease; CI, confidence interval; DSST, Digit Symbol Substitution Test; GCP, global cognitive performance.

## 4 Discussion

In this large, nationally representative cohort of older adults, we found that Ccr was strongly associated with cognitive performance across multiple domains, including memory and executive function, as assessed by CERAD, DSST, and Z‐GCP. Importantly, SIRI was found to partially mediate these associations, highlighting the pivotal role of systemic inflammation in the “kidney–brain” axis. These findings provide compelling evidence for a mechanistic link between renal function and cognition, emphasizing the clinical potential of Ccr and SIRI as accessible biomarkers for the early identification of individuals at elevated risk for cognitive decline.

The relationship between kidney function and neurocognitive health has long been recognized, yet the underlying mechanisms remain incompletely understood. Our findings demonstrated that reduced Ccr is closely associated with poorer performance in overall cognition, memory, and executive function, as assessed by the Z‐GCP, CERAD, and DSST, respectively. This domain‐specific vulnerability is consistent with previous longitudinal and cross‐sectional studies linking impaired kidney function to cognitive decline and increased dementia risk (Miller et al. 2022; Anand et al. 2014; Viggiano et al. 2020). From a neuroanatomical perspective, these cognitive domains depend on distinct yet highly vulnerable brain regions. The CERAD primarily evaluates episodic memory, which is critically dependent on the hippocampus and adjacent medial temporal lobe structures, particularly the entorhinal cortex and parahippocampal gyrus (Barry et al. 2021). Converging evidence from neuroimaging and neuropathological studies indicates that atrophy in these regions is closely related to impairments in episodic memory encoding and retrieval, representing a hallmark of early Alzheimer's disease pathology (Iglesias et al. 2016; Bayram et al. 2018). Voxel‐based morphometry and volumetric MRI studies further demonstrate that reduced volumes of the hippocampus and entorhinal cortex in individuals with mild cognitive impairment and Alzheimer's disease can predict lower immediate and delayed recall performance on the CERAD battery (Dos Santos et al. 2011). Additionally, the perforant pathway, a critical white matter tract connecting the entorhinal cortex and hippocampus, is essential for episodic memory. Diffusion tensor imaging and volumetric studies have demonstrated that degeneration of this pathway is closely associated with memory dysfunction (Rogalski et al. 2012).

In contrast, the DSST primarily assesses executive function, processing speed, and attention. Neuroimaging research has shown that successful DSST performance relies on the structural and functional integrity of the dorsolateral prefrontal cortex (DLPFC), fronto‐striatal circuits (particularly the caudate nucleus), parietal cortex, and associated white matter networks (Dyer et al. 2022; Nowrangi et al. 2014). The DLPFC plays a central role in task switching and processing speed, fronto‐striatal pathways are involved in response inhibition and goal‐directed behavior, and the parietal cortex supports attentional allocation and integration of complex information (He et al. 2022). Notably, in patients with declining renal function, executive dysfunction is prominent, and neuroimaging studies have revealed strong associations with increased white matter hyperintensities, small vessel disease, and fronto‐parietal atrophy (Auriel et al. 2016; Kim et al. 2022).

On a molecular level, the accumulation of uremic toxins such as guanidinosuccinate and indolephenol sulfate, secondary to impaired renal function, may inhibit mitochondrial respiratory chain complexes I and IV and impair α‐ketoglutarate dehydrogenase activity within the tricarboxylic acid cycle (Deckers et al. 2017; X. Chen et al. 2021; Calabrese et al. 2020). These disruptions result in impaired ATP synthesis at presynaptic terminals, particularly in neural circuits characterized by rapid neurotransmitter turnover. Furthermore, excess uremic toxins may overwhelm the compensatory capacity of the BBB, directly injure microvascular endothelial cells in the prefrontal cortex, and disrupt neuron–glia–vascular metabolic coupling (C. Y. Zhang et al. 2020; S. Wang et al. 2023; Choi et al. 2021). By contrast, no significant associations were observed for the AFT in fully adjusted models. This may be attributable to differences in dynamic neuroplasticity and compensatory reorganization between brain hemispheres. Language function, in particular, appears to benefit from cognitive reserve factors such as higher education and occupational complexity, rendering it more resistant to metabolic insult (Lee et al. 2022; Carapelle et al. 2020; Jin et al. 2023).

RCS analysis demonstrated a significant nonlinear association between Ccr and cognitive impairment on both the CERAD and DSST scales, with inflection points at 127.8 and 88.3 mL/min, respectively (*p* for nonlinearity < 0.05). This result indicates that the relationship between renal function and cognitive performance is not strictly linear. When Ccr falls below certain thresholds, even if it remains within the conventional normal range, the risk of cognitive impairment increases substantially. It is noteworthy that the lower threshold for DSST suggests that cognitive domains involving processing speed and executive function are especially sensitive to declines in renal function. This finding is consistent with previous research indicating that executive dysfunction is often an early manifestation of vascular cognitive impairment, and further suggests that even mild reductions in kidney function can negatively impact frontocortical circuits and higher‐order cognition (Golenia et al. 2023; Román 2003). Importantly, our findings highlight that small decreases in Ccr, even within the traditionally defined normal range, may indicate increased cognitive risk in older adults. Therefore, monitoring subtle changes in renal function could provide an early warning for cognitive impairment, supporting timely screening and intervention.

Subgroup analyses revealed substantial heterogeneity in the association between Ccr and cognitive function. The positive relationship was notably stronger in females, particularly for AFT and DSST, suggesting greater cognitive vulnerability among women with reduced renal function. This may reflect neuroprotective effects of estrogen, increased cerebrovascular reactivity, and sex‐specific health factors (Subramaniapillai et al. 2021; Arenaza‐Urquijo et al. 2024). Previous research has also reported that older women are at increased risk for vascular cognitive impairment (Szoeke et al. 2021). Age‐stratified analyses showed a stronger positive association between Ccr and cognitive performance in participants aged 70–80 years, with significant effect modification for AFT and Z‐GCP. These results suggest that age‐related declines in neural reserve, increased small vessel disease, and elevated systemic inflammation may exacerbate the cognitive impact of impaired renal function in older adults (Hernandez et al. 2024; Marseglia et al. 2025). Even mild renal declines in older adults may lead to more severe cognitive deficits, emphasizing the need for early screening and intervention in this high‐risk group. Chronic disease status significantly modified the Ccr–cognition relationship. In individuals without diabetes or hypertension, higher Ccr was positively associated with cognitive performance, suggesting that preserved metabolic and vascular health enhances kidney function's neuroprotective effects. Conversely, diabetes and hypertension appeared to attenuate these associations, likely due to microvascular injury, chronic inflammation, and oxidative stress (Lespinasse et al. 2023; Ji et al. 2023; Coutinho et al. 2017). Stroke history notably modified the Ccr–cognition relationship, with individuals who had cerebrovascular events showing greater neural vulnerability, making renal decline a stronger predictor of cognitive impairment (Tang et al. 2018). These findings indicate that the protective effect of Ccr on cognition is modulated by sex, age, and chronic conditions, supporting the need for individualized risk assessment and intervention. Future studies should investigate the biological mechanisms of the kidney–brain axis, including hormonal, inflammatory, and cerebrovascular factors, to inform targeted prevention strategies.

The study employed causal mediation modeling to examine the role of inflammatory pathways in the kidney–brain axis, revealing variability in SIRI's mediation effects across cognitive domains. SIRI mediated approximately 6.6% of CERAD, 3% of DSST, and 4.6% of Z‐GCP scores, with no significant effect on AFT. This suggests that inflammatory pathways impact cognitive domains specifically, potentially reflecting differences in neurophysiological mechanisms and brain region vulnerability. SIRI, integrating neutrophils, lymphocytes, and monocytes, may exacerbate white matter damage by activating microglia and promoting amyloid deposition, impairing processing speed and memory consolidation (Guo et al. 2024; X. Wang, Li, et al. 2022). CERAD and Z‐GCP primarily reflect the integrity of the fronto‐parietal network and hippocampus, which are sensitive to inflammation‐induced synaptic damage and BBB disruption (Weiner et al. 2009). Pro‐inflammatory factors like IL‐6 and TNF‐α activate microglia, accelerating white matter damage and amyloid deposition, leading to cognitive impairments (Beltran‐Velasco and Clemente‐Suárez 2025). Metabolic abnormalities and uremic toxins from renal dysfunction activate peripheral inflammation, affecting the central nervous system via BBB changes (Kim et al. 2022). Although SIRI's mediation effect was statistically significant, it was moderate, highlighting the complexity of the kidney–brain axis. CKD patients often have comorbid CVDs, hypertension, and metabolic disorders, which can independently or synergistically contribute to neurodegeneration (Bolignano et al. 2024; Drew et al. 2019). Genetic susceptibility, lifestyle, and comorbidities also influence cognitive outcomes, suggesting that future research should adopt a comprehensive multi‐pathway framework.

This study enhances methodological rigor by integrating multiple analytical approaches, thus strengthening causal inference and providing a reproducible framework for examining the complex relationships between biomarkers and cognitive function. However, several limitations must be considered. Primarily, the cross‐sectional design of NHANES inherently restricts causal interpretation. Despite extensive covariate adjustment, unmeasured variables such as physical activity, dietary patterns, sleep quality, and chronic psychological stress could contribute to residual confounding. The results are confined to US adults, limiting the generalizability to other ethnic and cultural populations. Notably, the study cohort demonstrated unusually high educational attainment (63% with college education or higher), suggesting potential selection or “healthy survivor” bias. However, given the established protective role of cognitive reserve in high‐education groups, the associations identified herein may arguably represent a conservative baseline relative to broader populations.

Furthermore, relying on a single baseline assessment captures only a static snapshot of renal and inflammatory profiles, potentially overlooking transient biological fluctuations. While SIRI is a robust composite index, the absence of concurrent data on classical inflammatory cytokines, such as IL‐6 and TNF‐α, limits a detailed mechanistic analysis of the inflammatory milieu. Finally, given that cognitive decline results from a complex interplay of factors beyond inflammation, including oxidative stress and vascular dysfunction, future research employing multi‐mediator models is necessary to unravel these intricate pathways.

Future research should validate and expand these findings through prospective, multicenter, and ethnically diverse cohort studies, clarifying the temporal and causal relationships between renal function, systemic inflammation, and cognitive outcomes. Integrating multi‐omics technologies (genomics, proteomics, metabolomics) with advanced neuroimaging will offer deeper insights into the molecular and structural foundations of the kidney–brain axis. Rigorous comparative analyses of SIRI and conventional inflammatory markers are crucial to assess its clinical applicability in risk stratification for cognitive impairment. Additionally, randomized controlled trials targeting modifiable factors, such as anti‐inflammatory interventions and lifestyle changes, are necessary to determine whether reducing systemic inflammation can prevent or delay cognitive decline in individuals with renal dysfunction. Ultimately, individualized risk prediction models and targeted therapeutic strategies offer promising approaches to improving cognitive health and quality of life in older adults at increased risk.

## 5 Conclusion

In summary, this study found that higher Ccr was independently associated with better cognitive performance and a lower risk of cognitive impairment among older adults, particularly in memory and executive function domains as assessed by CERAD, DSST, and Z‐GCP scores. Ccr showed predictive utility for cognitive impairment in ROC analysis, although the discriminatory power was moderate. Mediation analysis suggested that systemic inflammation, as measured by SIRI, may partially mediate the relationship between Ccr and cognitive function, indicating a potential contributory role of inflammation in cognitive decline among older adults. While these findings provide preliminary evidence supporting the relevance of renal health and systemic inflammation in cognitive aging, further prospective and mechanistic studies are needed to clarify causality and to evaluate their value in early risk identification and intervention.

## Author Contributions

**Yuyao He**: conceptualization, methodology, software, formal analysis, writing – original draft. **Wenyue Hu**: conceptualization, investigation, supervision, writing – review and editing. **Cheu Hiutung**: conceptualization, visualization, investigation, writing – review and editing. **Zhenyun Han**: funding acquisition. supervision, data curation, visualization, writing – review and editing.

## Funding

This work was supported by the Sanming Project of Medicine in Shenzhen (Grant No. SZZYSM202105010) and the National Natural Science Foundation of China (Grant No. 82274466).

## Ethics Statement

Research involving human participants was conducted in accordance with the Declaration of Helsinki. Ethical approval was obtained from the NHANES.

## Consent

All participants in the NHANES study provided written informed consent to the National Center for Health Statistics (NCHS). The present study utilized de‐identified, publicly available data from NHANES, and therefore, did not require additional informed consent from participants.

## Conflicts of Interest

The authors declare no conflicts of interest.

## Data Availability

The datasets analyzed during the current study are publicly available from the NHANES at https://wwwn.cdc.gov/nchs/nhanes/.
